# Foramen of Winslow hernia: a case report of a rare life-threatening situation

**DOI:** 10.1093/jscr/rjac364

**Published:** 2022-08-31

**Authors:** Ayoub Kharkhach, Andrea Police, Andrea Mabilia, Lionel Charre

**Affiliations:** Department of Viscéral Surgery, Faculty of Medicine and Pharmacy, Mohammed Ist University, Oujda, Morocco; Department of Visceral and Digestive Surgery, Simone Veil Hospital, Eaubonne, France; Department of Visceral and Digestive Surgery, Simone Veil Hospital, Eaubonne, France; Department of Visceral and Digestive Surgery, Simone Veil Hospital, Eaubonne, France

## Abstract

Internal hernias through the foramen of Winslow are considered as rare events. A 66-year-old female patient presented to our emergency department with epigastric and right upper quadrant pain associated with abdominal distention, nausea and vomiting and signs of shock. A computed tomography scan showed bowel strangulation with distended loops identified within the lesser sac. The diagnosis was confirmed by a midline laparotomy procedure. The ileum, the caecum and the ascending colon were found to herniate into the foramen of Winslow. A right hemicolectomy with a resection of the necrotic segment was performed. The patient recovered well postoperatively and was discharged on 10th day after surgery. The diagnosis and management of this rare disease remains challenging. Cross-sectional imaging with reconstruction is considered as the diagnostic modality of choice. Moreover, right hemicolectomy is a preferred procedure in order to decrease the rate of recurrence.

## INTRODUCTION

Internal hernias are protrusions of the abdominal viscera through a pre-existing intraperitoneal orifice. They are a common cause of acute intestinal obstruction with an ischemic component by vascular strangulation [1]. They have an incidence of 5.8% [2], and given their nonspecific clinical manifestations, a preoperative diagnosis can be difficult. There are many types of abdominal internal hernias, herein we will describe an extremely rare entity firstly described by Philipe Frédérique Blandin in 1823 [1], which is the foramen of Winslow hernia (FWH), in which small bowel loops, the terminal ileum, cecum and ascending colon, the transverse colon, gallbladder or omentum passes through the foramen of Winslow to the lesser sac of the peritoneal space [2]. In this paper, we report a new challenging case with a review of clinical signs, imaging procedures and therapeutic options.

## CASE REPORT

**Figure 1:**
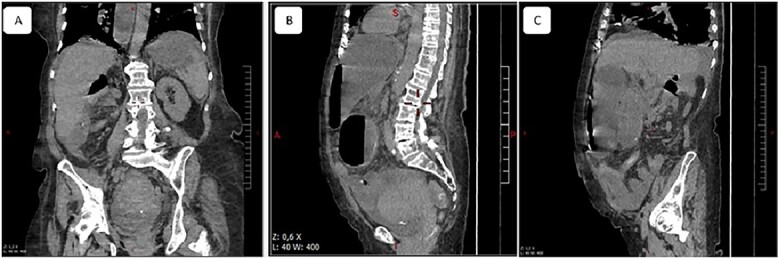
Multiple planes of computed tomography depicting the herniated cecum within the lesser sac. Reconstructed images: (**A**) axial, (**B**) and (**C**) sagittal views.

**Figure 2:**
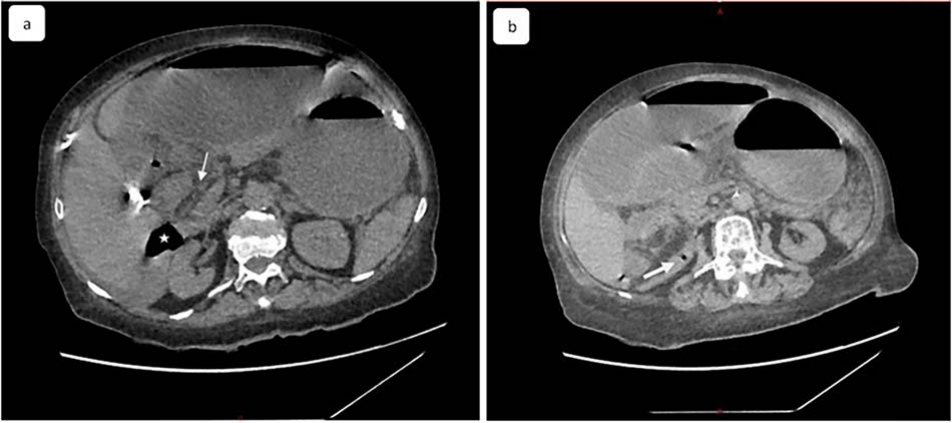
(**a**) Coronal view of computed tomography of abdomen and pelvis shows the dilated cecal pole and the stomach displaced anteriorly. Peritoneal fluid and some air bubbles related to intestinal perforation were observed in the lesser sac can also be observed (arrow head). (**b**) Closed-loop small bowel obstruction. CT scan shows a radial array of distented small bowel loops (asterisk) with stretched and thickened mesenteric vessels converging to a central point and anterior to inferior vena cava, which is compressed (white arrow).

**Figure 3:**
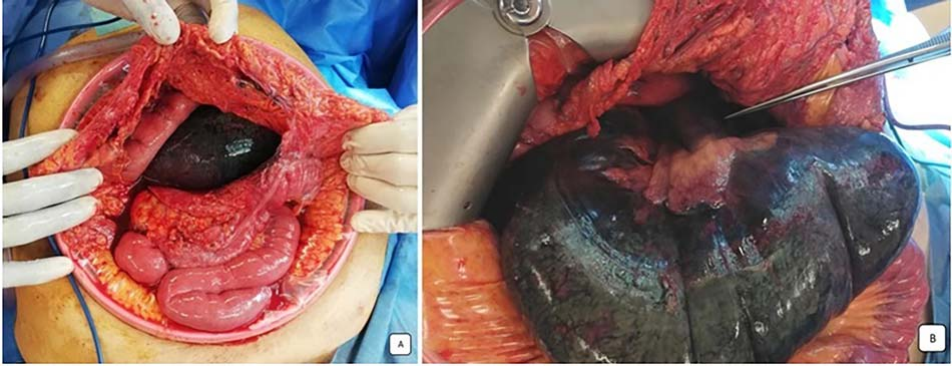
Peroperative images: (**A**) the distended and necrotic cecum after the opening of omental bursa by coloepiploicdetachement. (**B**) Ischemic herniated colon beside stretched loops and mesenteric vessels directed to the foramen of Winslow.

A 65-year-old woman presented to the emergency department with a complaint of acute abdominal pain and distention accompanied by nausea, vomiting and constipation of 2 days. She had a history of diabetes, hypothyroidism, dyslipidemia, mixed connective tissue disease and cholecystectomy. On examination, she had signs of septic shock with a hemodynamic instability, a Glasgow coma scale of 13 with generalized abdominal defense. Initial blood count showed elevated white blood cells (WBC) 22 000 elem/mm^3^ and C-reactive protein (CRP) of 500 mg/l and procalcitonin of 96 ng/ml. Her lactate on venous blood gas sampling doubled within an hour from 1.64 to 2.3 mmol/l. An urgent abdominal computed tomography (CT) was performed and revealed a dilated loop of bowel within the left upper quadrant and the lesser sac through the foramen of Winslow (Fig. 1). Furthermore, peritoneal fluid and some air bubbles related to intestinal perforation were observed in the omental bursa (Fig. 2). There was also mass effect on the hepatic hilum anteriorly and the inferior vena cava posteriorly. The stomach was pushed back antero-laterally with stretched mesenteric vessels directed to a central point (Fig. 3).

**Figure 4:**
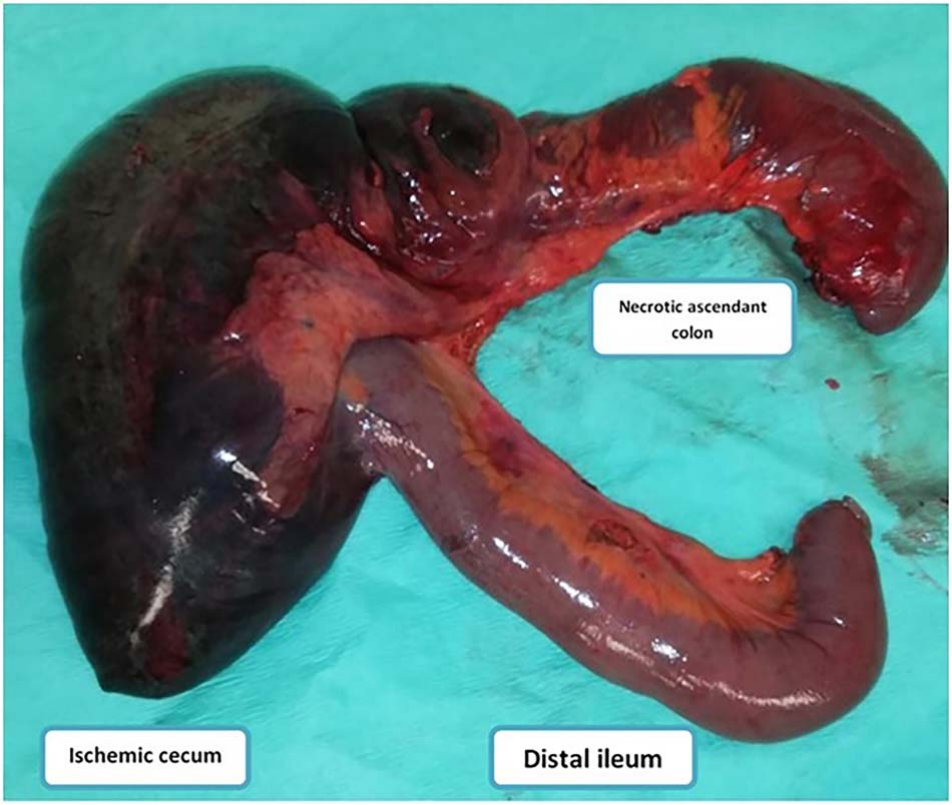
Image showing the incarcerated ileum and coecum after large surgical resection.

Our patient underwent an emergency laparotomy and the internal hernia was reduced manually after coloepiploic detachment. The herniated ileum and colon was found to be ischemic and was subsequently resected (Fig. 4) and a right double ileo-colostomy was performed. The histology of right colectomy showed lesions of acute ischemic colitis with hemorrhagic infarction of the ileocecal resection and appendix and no signs of malignancy were observed. The patient recovered well postoperatively with normalization of blood biology within several days. The stoma was viable and productive. She was discharged from the surgery department 10 days later and was referred to the medical department for the treatment of mixed connective tissue disease (MCTD) and died 2 months later from a severe pneumonia.

## DISCUSSION

FWHs are unknown, which may delay the diagnosis. This life-threatening surgical emergency has a rapid evolution and can lead to strangulation, gangrene of the bowel and peritonitis [2, 4]. Few cases have been reported in the literature to date with delayed diagnosis and necrosis. The most herniated viscus is small bowel and ileum in 70% of cases, followed by caecum or right colon. Resection and anastomosis or ileostomy formation was performed in all cases and the follow-up uneventful with a median of hospitalization of 9 days. The symptoms can range from mild digestive discomfort to an acute abdomen, and the severity may be associated with duration, reducibility of the hernia, the collar size and the presence or absence of strangulation. Therefore, vomiting is inconstant and the cessation of substances and gas is late [2, 7]. Biological assessment is not necessary for diagnosis, and may show elevated WBC and CRP associated with a high level of lactate in blood gas measurement and hydro-electrolytic disorders. However, a few cases of jaundice by compression of the hepatic pedicle [3, 7, 8] and one case of pancreatitis [4] have been described.

The most reported mechanisms implicated in the FWH pathogenesis are changes in the intra-abdominal pressure by pregnancy, immediate postprandial periods or overeating, distal obstructive lesions of the colon, in addition to, excessive viscera mobility with a ‘wandering cecum’ found at 10–20% of people, an abnormally long small bowel mesentery, the defects of the gastrohepatic ligaments or cholecystectomy [3, 5, 7, 9, 10].

Diagnosis of FWHs is made per-operatively in the majority of reported cases. Nevertheless, CT cross-sectional imaging with multiplanar reformation is considered the diagnostic modality of choice [3, 5, 7, 9, 10]. CT scan can identify the site and the cause of obstruction, the herniated segment and the presence of ischemic changes as lack of enhancement in the bowel involved. The characteristic imaging findings of FWHs are the following: The enlargement of the virtual space between the inferior vena cava and the portal vein with the abnormal presence of a distended closed-loop with stretched and twisted mesenteric vessels that converge with a ‘bird’s beak’ toward the foramen of Winslow [2, 3]. Other signs such as a decreased bowel wall enhancement, or focal discontinuity of the bowel wall with pneumoperitoneum or peritoneal fluid, should lead to a rapid laparotomy, as observed in our case. An image of the right iliac fossa recess with absence of the ascending colon in the right gutter and a directed cecum to the omental bursa may be suggestive of FWHs [1, 7].

Regarding the management of FWHs, the operative approach is the treatment of choice [5, 7, 11]. An exploratory surgery by laparotomy or laparoscopy, as advocated in several recent reports is a successful method that should be performed [3, 5, 7, 12]. The first operating stage is to reduce the hernia by traction. However, when the reduction is difficult, the opening the lesser sac, needle decompression or enterotomy of the closed-up loop, enlarging the foramen of Winslow by opening the gastrohepatic ligament, or even performing a Kocher’s maneuver may be necessary [1, 5]. Nonetheless, when signs of necrosis are present, it is vital to perform bowel resection, with anastomosis or double-stoma [1, 5].

Prophylactic measures to prevent recurrence are discussed such as the fixation of the hypermobile colon with cecopexy to the lateral abdominal wall, or, the closure of the foramen by simple stitches and mobilized great omentum [1, 5, 10, 11, 13]. Even though, the closure of the hiatus may have a negative sequel such as the damage of the hepatic pedicle or vein thrombosis as reported by Dorian *et al*. [6, 14]. To the best of our knowledge, there is no evidence on recurrent internal hernia in Winslow’s hiatus to date, and also, there are no definite criteria for these preventive strategies and their effectiveness.

To sum up, there is a consensus throughout the literature that ileo-colic resection is a good surgical solution allowing easy reduction of the hernia and reliable prevention of recurrence.

## CONCLUSION

FWH is a rare type of internal hernia with flat belly occlusion that is largely ignored by physicians and radiologists, which leads to a delay in diagnosis and may be dangerous for an acutely ill patient. The advent of CT with multiplanar reformation aid in making an early diagnosis and an appropriate surgery before ischemia occurs. Therapeutic strategies still needs to be established through larger cohort studies, yet, a prompt intervention allows uneventful recovery and reduce the morbidity and mortality rates of this uncommon disease.

## AUTHORS’ CONTRIBUTIONS

All authors contributed toward data analysis, drafting and revising the paper, gave final approval of the version to be published and agree to be accountable for all aspects of the work.

## CONFLICT OF INTEREST STATEMENT

The authors declare no conflict of interest.

## FUNDING

The author(s) received no financial support for the research, authorship and/or publication of this article.

## References

[1] Mathias J , PhiI Bruot O, Ganne PA, Laurent V, Regent D. Hernies internes. EMC (Elsevier Masson SAS, Paris) Radiodiagnostic-Appareildigestif, 33-015-A-37, 2008. 10.1016/S0246-0610(08)29704-5.

[2] Lanzetta MM, Masserelli A, Addeo G, Cozzi D, Maggialetti N, Danti G, Bartolini L, et al. Internal hernias: a difficult diagnostic challenge. Review of CT signs and clinical findings. Acta Biomed 2019;90:20–37. 10.23750/abm.v90i5-S.8344 PMC6625567 31085971

[3] Welaratne I , Nasoodi A. A rare cause of obstructive jaundice: cecal herniation through the foramen of Winslow. J Clin Imaging Science 2018;8:24. 10.4103/jcis.JCIS_12_18 PMC6029008 30034928

[4] Joo Y-E , Kim H-S, Choi S-K, Rew J-S, Kim H-R, Cho C-K, et al. Internal hernia presenting as obstructive jaundice and acute pancreatitis. Scand J Gastroenterol 2002;37:983–6. 12229977 10.1080/003655202760230982

[5] Moris D, Tsilimigras DI, Yerokun B, Seymour KA, Guerron AD, Fong PA, et al. Foramen of Winslow hernia: a review of the literature highlighting the role of laparoscopy. J Gastrointest Surg. 2019;23:2093–9. 10.1007/s11605-019-04353-3 31420858

[6] Doishita S, Takeshita T, Uchima Y, Kawasaki M, Shimono T, Yamashita A, et al. Internal hernias in the era of multidetector CT: correlation of imaging and surgical findings. Radiographics 2016;36:88–106. 26587890 10.1148/rg.2016150113

[7] Brunschweiler NA , Daoud M, Serot MC, Mauvais F. À propos d'un cas de hernie de l'angle iléocæcal à travers l'hiatus de Winslow - ScienceDirect. Ann Chir 2005;130:346–9. 15935793 10.1016/j.anchir.2005.02.012

[8] Duinhouwer LE , Deerenberg E, Rociu E, Kortekaas RT. Herniation of the colon through the foramen of Winslow-A case report. Int J Surg Case Rep 2016;24:14–7. 27176504 10.1016/j.ijscr.2016.04.044 PMC4874454

[9] Delamarre J , Henry X, Jouet-Gondry C, Daurat M, Deschepper B, Stoppa R. Cecal hernia through Winslow’s foramen. Radiographic study of a case and review of the literature. J Radiol 1988;69:459–64. 3047378

[9] Forbes SS, Stephen WJ. Herniation through the foramen of Winslow: radiographic and intraoperative findings. Canadian Journal of Surgery 2006;49:362+ PMC3207582 17152576

[10] Ozsoy M , Ozkececi T, Akici M, Kalkan M, Katirağ A, Yilmaz S. Internal herniation through the foramen of Winslow: a rare cause of bowel obstruction. ANZ J Surg 2015;87:E204–5. 10.1111/ans.13124 25913049

[11] Harnsberger CR , McLemore EC, Broderick RC, Fuchs HF, Yu PT, Berducci M, et al. Foramen of Winslow hernia: a minimally invasive approach. Surg Endosc 2015;29:2385–8. 25361659 10.1007/s00464-014-3944-5

[12] Sobek V , Chrostek M. Herniacecéka do foramen Winslowijakovzácnápříčinaileózníhostavu [Cecalherniationthrough the foramen of Winslow as a rare cause of ileus]. RozhlChir 2016;95:291–3. 27523178

[13] Garg S , Flumeri-Perez G, Perveen S, DeNoto G. Laparoscopic repair of foramen of Winslow hernia. Int J Angiol 2016;25:64–7. 26900313 10.1055/s-0033-1363497 PMC4758848

[14] Dorian AL , Stein GN. Hernia through foramen of Winslow. Report of a case with preoperative roentgen diagnostis and succesful surgical management. Surgery 1954;54:795–801. 13156898

